# Sensor kinase KinB and its pathway‐associated key factors sense the signal of nutrition starvation in sporulation of *Bacillus subtilis*

**DOI:** 10.1002/mbo3.566

**Published:** 2018-01-03

**Authors:** Weipeng Liu, Zeying He, Feng Gao, Jinyuan Yan, Xiaowei Huang

**Affiliations:** ^1^ State Key Laboratory for Conservation and Utilization of Bio‐Resources, and Key Laboratory for Microbial Resources of the Ministry of Education Yunnan University Kunming Yunnan China; ^2^ Yunnan Institute of Tropical Crops Jinghong Yunnan China; ^3^ Center Laboratory of the Second Hospital affiliated with Kunming Medical University Kunming China

**Keywords:** environmental signal, KapB, KbaA, nutrient starvation, sensor kinase KinB, signaling pathway, sporulation

## Abstract

*Bacillus subtilis* responds to environmental stress cues and develops endospores for survival. In the process of endospore formation, sporulation initiation is a vital stage and this stage is governed by autophosphorylation of the sensor histidine kinases. The second major sensor kinase KinB perceives the intracellular changes of GTP and ATP during sporulation. However, determination of the environmental signals as well as its related signaling pathway of KinB requires further elucidation. Our current study found that, contrary to the sporulation failure induced by Δ*kinA* in the nutrient‐rich 2× SG medium, the sensor kinase KinB sensed the environmental cues in the nutrient‐poor MM medium. Two other membrane proteins, KapB and KbaA, also responded similarly to the same external signal as KinB. Both KapB and KbaA acted upstream of KinB, but they exerted their regulation upon KinB independently. Furthermore, we demonstrated that both the SH3 domain and the α‐helix structure in KapB are required for sensing or transducing the signal of sporulation initiation. Collectively, our work here supplied the direct evidences that KinB and its pathway sense the external signal of nutrient starvation in MM medium, and further analyzes the interrelationship among KinB, KbaA, and KapB.

## 1 INTRODUCTION

Nutrient starvation or other adverse environmental conditions generally prompts *Bacillus subtilis* to form endospores for survival (Cano & Borucki, 1995). The change from vegetative growth to endospore formation represents a significant shift in life history for a unicellular bacterium to survive in hostile environments (Hall‐Stoodley, Costerton, & Stoodley, 2004; Stewart & Costerton, 2001). During this process, regulation of sporulation initiation is believed to be the most critical. The specific environmental cues stimulate autophosphorylation of the sensor kinases and then a phosphoryl group will be transferred through a multicomponent phosphorelay system (Fujita & Losick, 2005). Briefly, the activated sensor histidine kinase first phosphorylates the relay protein Spo0F into Spo0F~P. The phosphoryl group of Spo0F, in turn, is transferred to Spo0B. Subsequently, Spo0B donates the phosphoryl group to the key regulator Spo0A. As the intracellular activation of Spo0A~P reaches a certain threshold, it turns on transcription of those sporulation‐related genes (Burbulys, Trach, & Hoch, 1991; Fujita & Losick, 2005). The extracellular or intracellular signal activating the sensor histidine kinases is one of the earliest events for endospore formation.

In *B. subtilis*, the sensor kinases include at least five members, such as KinA–KinE, that sense different signals of sporulation (Jiang, Shao, Perego, & Hoch, 2000; LeDeaux, Yu, & Grossman, 1995; Piggot & Hilbert, 2004). Among them, KinA, as an intracellular sensor kinase having no protein domains outside the cell, has been suggested to respond to the shift in available ATP pool (Stephenson & Hoch, 2001). KinC senses the membrane damage as well as the leakage of potassium ions (López, 2015; López, Fischbach, Chu, Losick, & Kolter, 2009a; López, Gontang, & Kolter, 2010; López, Vlamakis, Losick, & Kolter, 2009b; Shemesh, Kolter, & Losick, 2010). KinD detects the signal of small molecule substances directly or indirectly (Aguilar, Vlamakis, Guzman, Losick, & Kolter, 2010; Chen et al., 2012; Wu et al., 2013; Zhang & Hendrickson, 2010). Besides regulating endospore formation, those five phosphorelay sensor kinases are also involved in multicellular behaviors such as biofilm formation and sliding motility, with each playing the overlapping but slightly different roles (Grau et al., 2015). But anyway, KinA and KinB are believed to be the major kinases for initiating sporulation.

KinB is the second major sensor kinase. During sporulation, a concurrent change of GTP decrease and ATP increase upregulates the transcriptions of *kinB* and *kinA*, which ultimately leads to the increase in Spo0A~P and the activation of the sigma cascade to produce endospores (Tojo, Hirooka, & Fujita, 2013). Structural analysis of KinB demonstrated that it was a membrane protein and composed of six transmembrane domains, a DHp domain, and a CA domain (Bick et al., 2004). Furthermore, the structure prediction indicated that KinB contained only loop regions and an N‐terminal segment as an extracellular sensor rather than an integral domain outside functioning as a sensor of extracellular signals (Parkinson & Kofoid, 1992). This characteristic of KinB implies that it is unlikely to receive extracellular signals directly, and some other membrane or membrane‐related proteins should be coupled with KinB (Dartois, Djavakhishvili, & Hoch, 1996; Phillips & Strauch, 2002). Indeed, it has been reported that another gene, *kapB*, was localized in the same operon as *kinB* using a single promoter. Their functions seemed to be related since the inactivation of *kapB* also led to a sporulation defect in the mutant strain MB340 (D*kinA96*) just as the KinB mutant did, suggesting that KapB plays a regulatory role in the expression of KinB or KapB is essential for the activation of KinB (Dartois, Djavakhishvili, & Hoch, 1997; Trach & Hoch, 1993). KbaA is another integral membrane protein containing six potential membrane‐spanning helices. It has been described that KbaA may execute a positive role to trigger the activation of KinB at the onset of sporulation (Dartois et al., 1996).

Although it has been described that the roles of proteins KbaA and KapB are coupled with KinB, most of the experimental evidences in this area were from the strain MB340 (D*kinA96*) that is a mutant strain with *kinA* deletion (Dartois et al., 1996, 1997; Trach & Hoch, 1993). However, whether the sensor kinases KinA and KinB, and the membrane proteins KapB and KbaA directly respond to the same environmental signals in sporulation or what is their interrelationship among KinB, KapB, and KbaA in the signaling pathway requires further elucidation. Our study showed the experimental evidences that KinB, KapB, and KbaA served in recognizing the environmental cues of nutrient starvation during sporulation. Both of the 2× SG and MM media are used to induce sporulation; compared to 2× SG medium, the latter usually simulates the nutrient starvation environment. So in the nutrient‐poor MM medium, disruption of the sensor kinase KinB, instead of KinA, would lead to serious defects on sporulation. As to the signaling pathway, our data indicated that KapB and KbaA function upstream of KinB and jointly regulated KinB. Furthermore, we demonstrated that the SH3 domain and the α‐helix structure of KapB protein were essential during sporulation.

## 2 MATERIALS AND METHODS

### 2.1 Bacterial strains, plasmids, and media

The strains of *B. subtilis* and *Escherichia coli* as well as the plasmids used in this study are listed in Table 1. All *B. subtilis* strains were the derivatives of *B. subtilis* 168 strain via transformation with plasmid DNAs. The mutant strains with gene deletion were verified by PCR analysis and DNA sequencing. The primers used for mutant construction or verification are listed in Table 2.

**Table 1:** Strains and plasmids used in this study

Strain or plasmid	Genotype/description	Source or reference
Strains		
*Bacillus subtilis* 168	Wild type	From Bacillus Genetic Stock Center
Δ*kinB*	Δ*kinB*; erm	This work
Δ*kinA*	Δ*kinA*; cm	Gift of Kazuo Kobayashi
P*spoIIG‐lacZ*	*amyE*::P*spoIIG‐lacZ*, reporter	This work
P*spoIIG‐lacZ* Δ*kinB*	Δ*kinB*; erm; *amyE*::P*spoIIG‐lacZ*, reporter	This work
P*spoIIG‐lacZ* Δ*kinA*	Δ*kinA*; cm; *amyE*::P*spoIIG‐lacZ*, reporter	This work
Δ*kinB*Δ*kapB*	Δ*kinB*; erm; Δ*kapB*; cm	This work
Δ*kbaA*	Δ*kbaA*; cm	This work
Δ*kinB*Δ*kbaA*	Δ*kinB*; erm; Δ*kbaA*; cm	This work
Δ*kapB*Δ*kbaA*	Δ*kapB*; cm; Δ*kbaA*; cm	This work
pDG148‐*kapB*Δ*kinB* Δ*kapB*	Δ*kinB*; erm; Δ*kapB*; cm; pDG148‐*kapB*	This work
pDG148‐*kbaA*Δ*kinB* Δ*kbaA*	Δ*kinB*; erm; Δ*kbaA*; cm; pDG148‐*kbaA*	This work
pDG148‐*kinB* Δ*kinB* Δ*kapB*	Δ*kinB*; erm; Δ*kapB*; cm; pDG148‐*kinB*	This work
pDG148‐*kinB* Δ*kinB* Δ*kbaA*	Δ*kinB*; erm; Δ*kbaA*; cm; pDG148‐*kinB*	This work
P*spoIIG‐lacZ* Δ*kapB*	Δ*kapB*; erm; *amyE*::P*spoIIG‐lacZ*, reporter	This work
P*spoIIG‐lacZ* Δ*kinB* Δ*kapB*	Δ*kinB*; erm; Δ*kapB*; cm; *amyE*::P*spoIIG‐lacZ*, reporter	This work
P*spoIIG‐lacZ* Δ*kbaA*	Δ*kbaA*; cm; *amyE*::P*spoIIG‐lacZ*, reporter	This work
P*spoIIG‐lacZ* Δ*kinB* Δ*kbaA*	Δ*kinB*; erm; Δ*kbaA*; cm; *amyE*::P*spoIIG‐lacZ*, reporter	This work
pDG148‐*kapB* P*spoIIG‐lacZ* Δ*kinB* Δ*kapB*	Δ*kinB*; erm; Δ*kapB*; cm; pDG148‐*kapB*;* amyE*::P*spoIIG‐lacZ*, reporter	This work
pDG148‐*kinB* P*spoIIG‐lacZ* Δ*kinB* Δ*kapB*	Δ*kinB*; erm; Δ*kapB*; cm; pDG148‐*kinB*;* amyE*::P*spoIIG‐lacZ*, reporter	This work
pDG148‐*kbaA* P*spoIIG‐lacZ* Δ*kinB* Δ*kbaA*	Δ*kinB*; erm; Δ*kbaA*; cm; pDG148‐*kbaA*;* amyE*::P*spoIIG‐lacZ*, reporter	This work
pDG148‐*kinB* P*spoIIG‐lacZ* Δ*kinB* Δ*kbaA*	Δ*kinB*; erm; Δ*kbaA*; cm; pDG148‐*kinB*;* amyE*::P*spoIIG‐lacZ*, reporter	This work
pDG148‐*kapB* Δ*kapB*	Δ*kapB*; cm; pDG148‐*kapB*	This work
pDG148‐*kapB*(1–80) Δ*kapB*	Δ*kapB*; cm; pDG148‐*kapB*(1–80)	This work
pDG148‐*kapB*(41–124) Δ*kapB*	Δ*kapB*; cm; pDG148‐*kapB*(41–124)	This work
pDG148‐*kapB* P*spoIIG‐lacZ* Δ*kapB*	Δ*kapB*; cm; pDG148‐*kapB*;* amyE*::P*spoIIG‐lacZ*, reporter	This work
pDG148‐*kapB* (1–80) P*spoIIG‐lacZ* Δ*kapB*	Δ*kapB*; cm; pDG148‐*kapB*(1–80); *amyE*::P*spoIIG‐lacZ*, reporter	This work
pDG148‐*kapB*(41–124) P*spoIIG‐lacZ* Δ*kapB*	Δ*kapB*; cm; pDG148‐*kapB*(41–124); *amyE*::P*spoIIG‐lacZ*, reporter	This work
Plasmids		
*p*DG1728	Bla, erm, spc, spoVG‐lacZ,amyE, Pspac	Bacillus Genetic Stock Center
*p*SPOIIG	Amp, spc, spoIIG‐lacZ	Synthesized by Shanghai Generay Co.
*p*DG148	Kan, amp, lacI, phl,	Bacillus Genetic Stock Center
*p*EASY™‐T5	Ppen, Pspac, Amp, kan	TransGen Biotech, China
*p*MD19‐T	Amp	TaKaRa
*p*CP115	Amp	Bacillus Genetic Stock Center
*p*Δ*kinB*	Erm, *kinB*	This work
*p*Δ*kapB*	Cm, *kapB*	This work
*p*Δ*kbaA*	Cm, *kbaA*	This work
*pDG148‐kapB*	Kan, amp, *kapB*	This work
*pDG148‐kinB*	Kan, amp, *kinB*	This work
*pDG148‐kbaA*	Kan, amp, *kbaA*	This work
*pDG148‐kapB* (1–80)	Kan, amp, *kapB*(1–80)	This work
*pDG148‐kapB* (41–124)	Kan, amp, *kapB*(41–124)	This work

**Table 2:** The oligonucleotide primers used in this study

Sequence (5′–3′)	Function and source
TCAAAGCCGCATTATCGTACATTTCCGTGTCGCCCTTGCAGCTGATCAATAAAGAC	Upstream primer for 5′ flanking *kinB* gene Downstream primer for 5′ flanking *kinB* gene
GTCTTTATTGAGCTGCAAGGGCGACACGGAAATGTGATAAGGGCAGATAGGTACAAAAGCGACTCATAGA	Upstream primer for erythromycin resistant geneDownstream primer for erythromycin resistant gene
TCTATGAGTCGCTTTTGTACCTATCTGCCCTTATCACCTGCAAGCCGTGATTTCT	Upstream primer for 3′ flanking *kinB* geneDownstream primer for 3′ flanking *kinB* gene
AAGCTT ACATTTTGAAGCGGGACAGGATCC AGCCAGTCCTCATCAT	Upstream primer for *kapB* gene‐knockoutDownstream primer for *kapB* gene‐knockout
AAGCTTTAACATTTTGAAGCGGGACAGGATCCTAAAGCCAGTCCTCATCAT	Upstream primer for *kbaA* gene‐knockoutDownstream primer for *kbaA* gene‐knockout
GTCGACATGAAAAGCCGTGGTTTAGTTCGCATGCTTACTTTGCTGCAAACTTCG	Upstream primer for *KapB* overexpressionDownstream primer for *KapB* overexpression
GTCGACATGAGCACGTTTGAGACAGGCATGCTTATGGCCGGTTAAAATATTC	Upstream primer for *KbaA* overexpressionDownstream primer for *KbaA* overexpression
GTCGACATGGAGATTGTAAAGGATTACGCATGCCTAGTGATGGTGATGATGGTGAACAG	Upstream primer for *KinB* overexpressionDownstream primer for *KinB* overexpression
CTTCTGGAATCTGGATCCCTGCACTATCAACACACTCTTCCCGCTTCAAATGTTAG	Upstream primer for 5′ flanking *kbaA* gene in Δ*kbaA*Δ*kapB*Downstream primer for 5′ flanking *kbaA* gene in Δ*kbaA*Δ*kapB*
CTAACATTTGAAGCGGGGAGTGTGTTGATAGTGCAGGCCAGTCCTCATTTACTTATTTCCTCCCGTTAAA	Upstream primer for erythromycin resistant gene in Δ*kbaA*Δ*kapB*Downstream primer for erythromycin resistant gene in Δ*kbaA*Δ*kapB*
TTTAACGGGAGGAAATAAGTAAATGATGAGGACTGGCGACATACAGCTATTAAGACCC	Upstream primer for 3′ flanking *kbaA* gene in Δ*kbaA*Δ*kapB*Downstream primer for 3′ flanking *kbaA* gene in Δ*kbaA*Δ*kapB*
GTCGACCATCCCGCTCAGGGAGACGCATGCTTAGGCCGGTTAAAATATTC	Upstream primer for expressing *KapB* (1–80)Downstream primer for expressing *KapB* (1–80)
GTCGACATGAGCACGTTTGAGACAGGCTGCATGCATAAGGCTTCACCATATGG	Upstream primer for expressing *KapB* (41–124)Downstream primer for expressing *KapB* (41–124)

*Bacillus subtilis* 168 and its derivatives were grown in Luria–Bertani (LB) broth at 37°C overnight for propagation. Sporulation was induced in 2× SG medium, a modified Schaeffer's medium containing 0.3 g/L beef extract, 0.5 g/L peptone, 0.5 g/L MgSO_4_·7H_2_O, 2.0 g/L KCl, 100 μmol/L MnCl_2_, 1 mmol/L Ca(NO_3_)_2_, 1 μmol/L FeSO_4_·7H_2_O, and 0.1% glucose (Leighton & Doi, 1971). To simulate the nutrient starvation environment, cells were induced for sporulation in MM medium containing 0.106 g/L K_3_PO_4_, 0.132 g/L (NH_4_)_2_SO_4_, 1.046 g/L MOPS, 0.588 g/L sodium citrate, 0.04 g/L MgCl_2_·6H_2_O, 0.04 g/L l‐tryptophan, 0.04 g/L l‐methionine, 0.7 mmol/L CaCl_2_·2H_2_O, 50 μmol/L MnCl_2_·4H_2_O, 5 μmol/L FeCl_3_·6H_2_O, 1 μmol/L ZnCl_3_, 2 μmol/L VB1, and 0.1% glucose. The antibiotics were added at the following concentrations accordingly: 5 μg/ml chloramphenicol, 5 μg/ml kanamycin, 100 μg/ml spectinomycin, or 1 μg/ml erythromycin.

### 2.2 Genetic manipulation

A method of double crossover homologous recombination was used to construct Δ*kinB* mutant of *B. subtilis*. Two flanking fragments of *kinB* gene and the erythromycin‐resistant gene were amplified via PCR, using the genome of *B. subtilis* 168 or the plasmid of pMG36e as templates, respectively. Those three fragments obtained from PCR were linked by overlapping PCR, and the newly connected fragment was then inserted into a pEASY™‐T5 vector to obtain the recombinant plasmid *p*Δ*kinB*. The plasmid *p*Δ*kinB* was transformed into *B. subtilis* 168 to generate the Δ*kinB* mutant strain.

The integration vector *p*CP115 was used to construct a knockout mutation of Δ*kapB* strain. Similarly, the homologous fragment of *kapB* gene was amplified via PCR. The PCR product was digested with *Hind*III and *Sph*I at primer‐incorporated restriction sites and inserted into a *Hind*III/*Sph*I digested *p*CP115 vector to obtain the recombinant plasmid *p*Δ*kapB*, which was then transformed into *B. subtilis* 168 to obtain the Δ*kapB* mutant. The knockout plasmid *p*Δ*kbaA* and the Δ*kbaA* mutant were obtained in the same way as *p*Δ*kapB* and the Δ*kapB* mutant. Additionally, the recombinant plasmids *p*Δ*kapB* and *p*Δ*kbaA* were also transformed into the Δ*kinB* mutant strain to obtain the double mutants Δ*kinB*Δ*kapB* and Δ*kinB*Δ*kbaA*, respectively.

To complement the expression of KapB, KbaA, and KinB, the full encoding regions of those genes were amplified via PCR. The PCR products were digested with *SalI* and *Sph*I and cloned into the corresponding sites of *pDG148* vector to obtain the plasmids of *pDG148‐kapB*,* pDG148‐kbaA*,* pDG148‐kinB*. Those three recombinant plasmids were then transformed into the Δ*kinB*Δ*kapB* and Δ*kinB*Δ*kbaA* strains to achieve the complement expressions of KapB, KbaA, and KinB in the different strains. The empty vector *p*DG148 was also transformed into the same bacterial strains using as the blank control.

For the construction of Δ*kbaA*Δ*kapB* mutant, two flanking fragments of the *kbaA* gene and the erythromycin‐resistant gene were amplified via PCR, connected by overlapping PCR, and inserted into a *pEASY*‐T5 vector. The constructed plasmid was finally transformed into Δ*kapB* mutant to obtain the double mutant of Δ*kapB*Δ*kbaA*.

### 2.3 Assay for growth kinetics

Growth rates of different strains, including Δ*kinA*, Δ*kinB*, Δ*kbaA*, Δ*kapB*, and wild‐type strain *B. subtilis* 168, in LB and MM media were determined. One ml of overnight culture of each strain was added to 100 ml of fresh LB and MM medium, and the optical density (OD600) was measured every 2 hr for a growth kinetic. The data for each strain was collected from at least three biological replicas to determine their growth rates.

### 2.4 Sporulation assays

To determine the efficiencies of sporulation, the bacterial strains were first incubated in a shaker in LB broth at 37°C for 8–10 hr. After centrifugation, all the precipitated bacterial cells in cell cultures were spotted into 2× SG or MM medium to induce sporulation. Spores were analyzed at 24 hr and 36 hr post inoculation. After the vegetative cells were killed at 80°C for 15 min, the viable cells representing spores were measured by plating onto LB agar medium. The viable cells per milliliter from each sample were also counted before the heat treatment as the total CFU. Sporulation frequency is determined by the ratio of spore number per milliliter to the total CFU (LeDeaux et al., 1995). The data for each strain was collected from at least three biological replicas.

### 2.5 β‐Galactosidase assays

To analyze the expression of *spoIIG*, the promoter region of *spoIIG* was fused to *p*DG1728 that contained the reporter gene *lacZ*. The *p*spoIIG reporter plasmid was successfully constructed by Shanghai Generay Biotech Co. Ltd. Then the reporter plasmid was transformed into the wild‐type strain *B. subtilis* 168 as well as the other mutants, respectively.

After cultivating in 2× SG or MM medium at 37°C for 24 hr to induce sporulation, the strains containing *lacZ* fusions were analyzed for β‐galactosidase activities as previously reported (Ferrari, Henner, Perego, & Hoch, 1988). Briefly, the activities were assessed with *ο*‐nitrophenyl‐β‐d‐galactopyranoside as a substrate and were expressed in Miller units. All assays were repeated at least three times for each strain.

### 2.6 Prediction of protein domain boundaries

Structural prediction of KapB was carried out at http://smart.embl-heidelberg.de/ (Schultz, Milpetz, Bork, & Ponting, 1998). Based on the results of online prediction, we constructed two vectors to overexpress the mutant proteins of KapB, including one protein without α‐helix (1–240 nt) and the other without SH3 domain (120–387 nt). The amplified gene fragments were digested with *Sal*I and *Sph*I and cloned into the plasmid *p*DG148 to obtain plasmids *pDG148‐kapB*(1–80) and *pDG148‐kapB*(41–124), respectively. These two recombinant plasmids above, as well as the blank vector *p*DG148, were all transformed into the Δ*kapB* mutant.

### 2.7 Western blotting

The bacterial strains were grown in LB broth at 37°C to an optical density of OD_600_ 0.6, at which point isopropyl β‐d‐1‐thiogalactopyranoside (IPTG) was added to a final concentration of 1 mmol/L. After continuous shaking 8 hr, the cells were centrifuged and washed twice with the ice‐cold phosphate‐buffered saline (PBS). The total protein samples from bacterial cells were extracted using the Bacterial Protein Extraction Kit from Sangon (Shanghai, China). After the protein concentration was determined using a microbicinchoninic acid (BCA) assay (Beyotime Biotechnology, Shanghai, China), 30 μg proteins per lane were separated by 15% SDS‐PAGE and transferred to PVDF membranes. The membranes were blocked in Tris‐buffered saline mixed with Tween‐20 (TBST, pH 7.4) containing 5% skim milk for 1 hr. Because the expressed proteins were fused with the 6× His tags, the membranes were then incubated with rabbit polyclonal anti‐6× His tag antibody (GeneTex, USA) in blocking buffer at 4°C overnight. After rinsing with TBST, the blots were incubated with the appropriate HRP‐conjugated secondary antibody for 4 hr and visualized using an enhanced chemiluminescence detection system as recommended by the manufacturer (Millipore, Billerica, MA, USA).

### 2.8 Statistical analysis

All data were calculated and expressed as the mean ± standard deviation (*SD*) before statistical analyses. Statistical comparisons were performed by a one‐way analysis of variance (ANOVA) followed by Dunnett's *t* test.

## 3 RESULTS

### 3.1 KinB is involved in sensing nutrient starvation in MM medium to initiate sporulation

**Figure 1:**
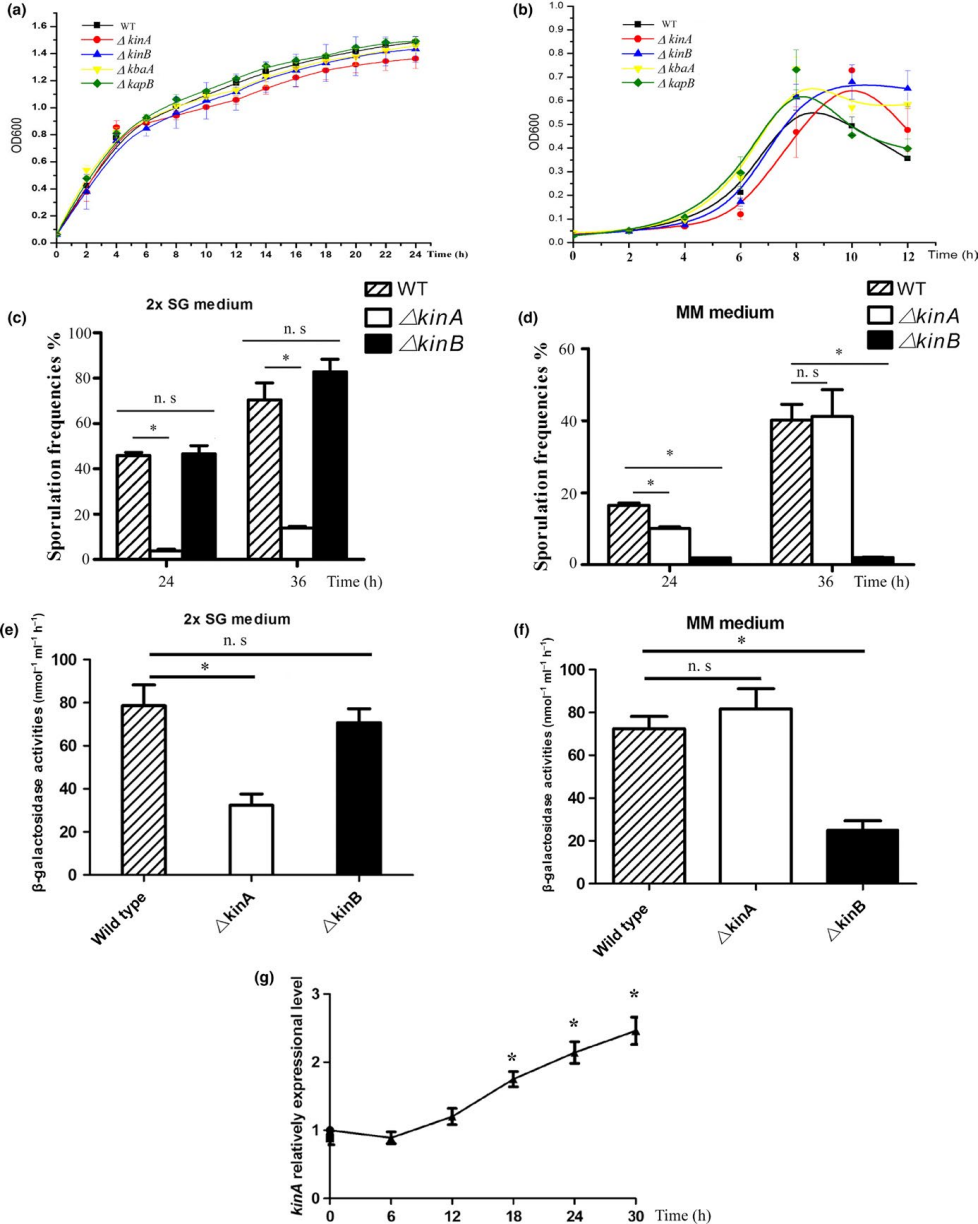
KinB is a more important sensor than KinA in response to the external cues of nutrient starvation in MM medium. (a, b) The growth curves of wild‐type, Δ*kinA*, Δ*kinB*, Δ*kapB*, and Δ*kbaA* in Luria–Bertani (LB) and MM media. All the tested mutants and the wild‐type strain showed similar growths in LB media, while they had different growths in MM media. (c, d) Comparing the sporulation frequencies of Δ*kinA*, Δ*kinB*, and the wild‐type *Bacillus subtilis* 168 in nutrition‐rich 2× SG medium and nutrition‐poor MM medium, respectively. Contrary to the sporulation failure due to Δ*kinA* mutation in the nutrient‐rich 2× SG medium, disruption of *kinB* led to a serious sporulation defect only in MM medium. (e, f) β‐Galactosidase activities of Δ*kinA*, Δ*kinB*, and the wild‐type *B. subtilis* 168 in nutrition‐rich 2× SG medium and nutrition‐poor MM medium after growing for 36 hr, respectively. Compared to the Δ*kinA* mutant and the wild‐type strain, Δ*kinB* had significantly decreased β‐galactosidase activity in MM medium. (g) The experiment of qPCR determined the relative expressional level of *kinA* in MM medium. not significant *p *≥ .05, *p < .05

To differentiate the environmental signals that the two major sensor kinases KinA and KinB perceive during sporulation initiation, we compared the frequencies of sporulation of △*kinB* with the wild‐type and △*kinA* strains after they were each placed in both the nutrient‐rich 2× SG medium and the nutrient‐poor MM medium. Because the different strains may have the different growth rates and the growth rates can sequentially influence sporulation rates, we analyzed their growth rates before the sporulation frequencies were determined. In growth kinetics of the wild‐type strain *B. subtilis* 168 and Δ*kinA* and Δ*kinB* mutants, it was shown that all displayed similar growth rates in LB medium and the cells reached their stationary phase within 12 hr (Figure 1a). However, in the nutrient‐poor MM medium, the cell densities of *B. subtilis* 168, Δ*kinA*, and Δ*kinB* were much lower than those in LB medium, and their growth rates were more variable (Figure 1b). Thus, in our sporulation assay, the tested bacterial strains were first propagated in LB broth at 37°C for 8–10 hr, and after the cultures were washed with 2× SG or MM medium, all cells were then spotted into the same fresh medium to induce sporulation. Our results of sporulation demonstrated that, comparing with the wild‐type strain (45.8 ± 1.5% and 70.4 ± 7.5%), the sporulation frequency of the Δ*kinA* mutant (3.8 ± 0.8% and 13.9 ± 0.7%) dropped significantly at either 24 hr or 36 hr in 2× SG medium (*p *< .05), but no significant difference was observed between the Δ*kinB* mutant (46.7 ± 3.6% and 82.9 ± 5.3%) and the wild‐type strain at the same time points (*p* ≥ .05) (Figure 1c). However, when the frequencies of sporulation of Δ*kinA* and Δ*kinB* mutants were determined again in MM medium, the results were opposite: the sporulation frequency in the Δ*kinB* mutant (1.9 ± 0.0% and 2.0 ± 0.1%) decreased sharply at either 24 hr or 36 hr (*p* < .05); Δ*kinA* (41.2 ± 7.3%) retained the similar capability for forming endospore at 36 hr to the wild‐type strain (40.2 ± 4.4%) (*p* ≥ .05) (Figure 1d). These results suggest that, between the most common sensor kinases KinA and KinB, the latter preferentially responds to the environmental cues of nutrient starvation in MM medium directly or indirectly during sporulation.

To further verify our hypothesis that KinB was more responsible for sensing nutrient starvation in MM medium and to activate the downstream sporulation‐related genes, the promoter region of *spoIIG*, a gene known to be under the direct control of Spo0A (Satola, Baldus, & Moran, 1992), was cloned and fused to the reporter plasmid *p*DG1728 to drive the expression of β‐galactosidase to show the activation of phosphorelay system during sporulation initiation. Altogether, three reporter strains were successfully constructed by transforming the recombinant plasmid into the wild‐type strain *B. subtilis* 168, Δ*kinA*, and Δ*kinB*, respectively. After analyzing their β‐galactosidase activities, it was found that compared to the wild‐type strain (78.6 ± 9.7 nmol/ml/hr), the β‐galactosidase activity in the Δ*kinA* mutant was reduced significantly (*p* < .05), about half of the wild‐type strain at 24 hr in 2× SG medium (32.4 ± 5.2 nmol/ml/hr). Contrarily, little difference was observed in MM medium since Δ*kinA* had a similar activity of β‐galactosidase (81.7 ± 9.4 nmol/ml/hr) as *B. subtilis* 168 (72.4 ± 5.7 nmol/ml/hr) (*p* ≥ .05) (Figure 1e). As for the Δ*kinB* mutant, the β‐galactosidase activity (25.1 ± 4.3 nmol/ml/hr) decreased significantly in MM medium (*p* < .05), but remained comparable to wild‐type strain in 2× SG medium (70.8 ± 6.4 nmol/ml/hr) (*p* ≥ .05) (Figure 1f).

Meanwhile, qPCR experiment was employed to determine the expressional level of *kinA* after transferred into MM medium. The result showed that the expression of *kinA* could be induced to more than twofold after 30 hr (Figure 1g). Collectively, the results above suggest that KinB played a role in perceiving the environmental signal in the MM medium though the other histidine kinase KinA retained its normal expression synchronously.

### 3.2 KapB and KbaA respond to the same environmental cues in MM medium

**Figure 2:**
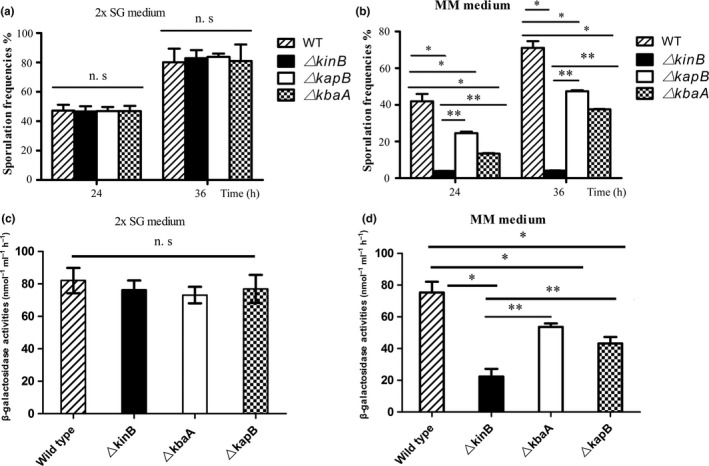
The integral membrane protein KbaA and the membrane lipoprotein KapB coupled with KinB and function as the major signaling pathway of sporulation in MM medium. (a, b) Comparing the sporulation frequencies of Δ*kinB*, Δ*kbaA*, and Δ*kapB* mutants and the wild‐type *Bacillus subtilis* 168 in nutrition‐rich 2× SG medium and nutrition‐poor MM medium, respectively. Similar to the Δ*kinB* mutant, the deletion of genes *kbaA* and *kapB* led to sporulation failure in MM medium. (c, d) β‐Galactosidase activities of Δ*kinB*, Δ*kbaA*, and Δ*kapB* mutants and the wild‐type *B. subtilis* 168 in nutrition‐rich 2× SG medium and nutrition‐poor MM medium after growing for 36 hr, respectively. The mutants of Δ*kbaA* and Δ*kapB* represented reduced β‐galactosidase activities in MM medium. n.s. *p *≥* *.05, *p < .05 using the wild‐type strain *B. subtilis* 168 as control, **p < .05 using the mutant strains △*kinB* as control

It had been suggested that the functions of the integral membrane protein KbaA and the membrane lipoprotein KapB should be coupled with the histidine kinase KinB (Dartois et al., 1996, 1997). Therefore, based on our experimental data above, we speculated that KapB and KbaA should have the similar phenotype with KinB in MM medium. To verify our hypothesis, we compared the sporulation frequencies and β**‐**galactosidase activities in the mutants of Δ*kinB*, Δ*kbaA*, and Δ*kapB*, respectively. Our results of sporulation frequency showed that, when induced in the nutrient‐poor MM medium, the sporulation frequencies in Δ*kbaA* (6.7 ± 0.2% at 24 hr and 18.8 ± 0.1% at 36 hr) and Δ*kapB* (12.3 ± 0.4% at 24 hr and 23.7 ± 0.3% at 36 hr) were much lower than that of the wild‐type *B. subtilis* 168 (20.9 ± 2.0% at 24 hr and 35.6 ± 1.8% at 36 hr) (*p* < .05) (Figure 2b). But either Δ*kbaA* or Δ*kapB* had relatively higher sporulation frequencies than Δ*kinB* (1.9 ± 0.0% at 24 hr and 2.7 ± 0.0% at 36 hr) (*p* < .05) (Figure 2b). When sporulation of all the strains were determined in the nutrient‐rich 2× SG medium, no distinct differences in sporulation frequencies were detected among Δ*kbaA* (46.8 ± 3.6% at 24 hr and 81.0 ± 11.2% at 36 hr), Δ*kapB* (46.8 ± 2.9% at 24 hr and 83.8 ± 2.3% at 36 hr), Δ*kinB* (46.7 ± 3.6% at 24 hr and 82.9 ± 5.5% at 36 hr), or the wild‐type *B. subtilis* 168 (47.2 ± 4.0% at 24 hr and 80.1 ± 9.2% at 36 hr) (*p* ≥ .05) (Figure 2a). Furthermore, the results from β‐galactosidase assays were consistent with those of sporulation (Figure 2c,d).

### 3.3 KapB and KbaA function upstream and regulate KinB independently

To explore the regulatory relationships among KbaA, KapB, and KinB in the signaling pathway, two double mutants of Δ*kinB*Δ*kbaA* and Δ*kinB*Δ*kapB* as well as a series of mutant strains with the complementary expressions, including *p*DG148‐*kinB*Δ*kinB*Δ*kbaA*,* p*DG148‐*kbaA*Δ*kinB*Δ*kbaA*,* p*DG148‐*kinB*Δ*kinB*Δ*kapB*, and *p*DG148‐*kapB*Δ*kinB*Δ*kapB*, were also constructed and analyzed.

**Figure 3:**
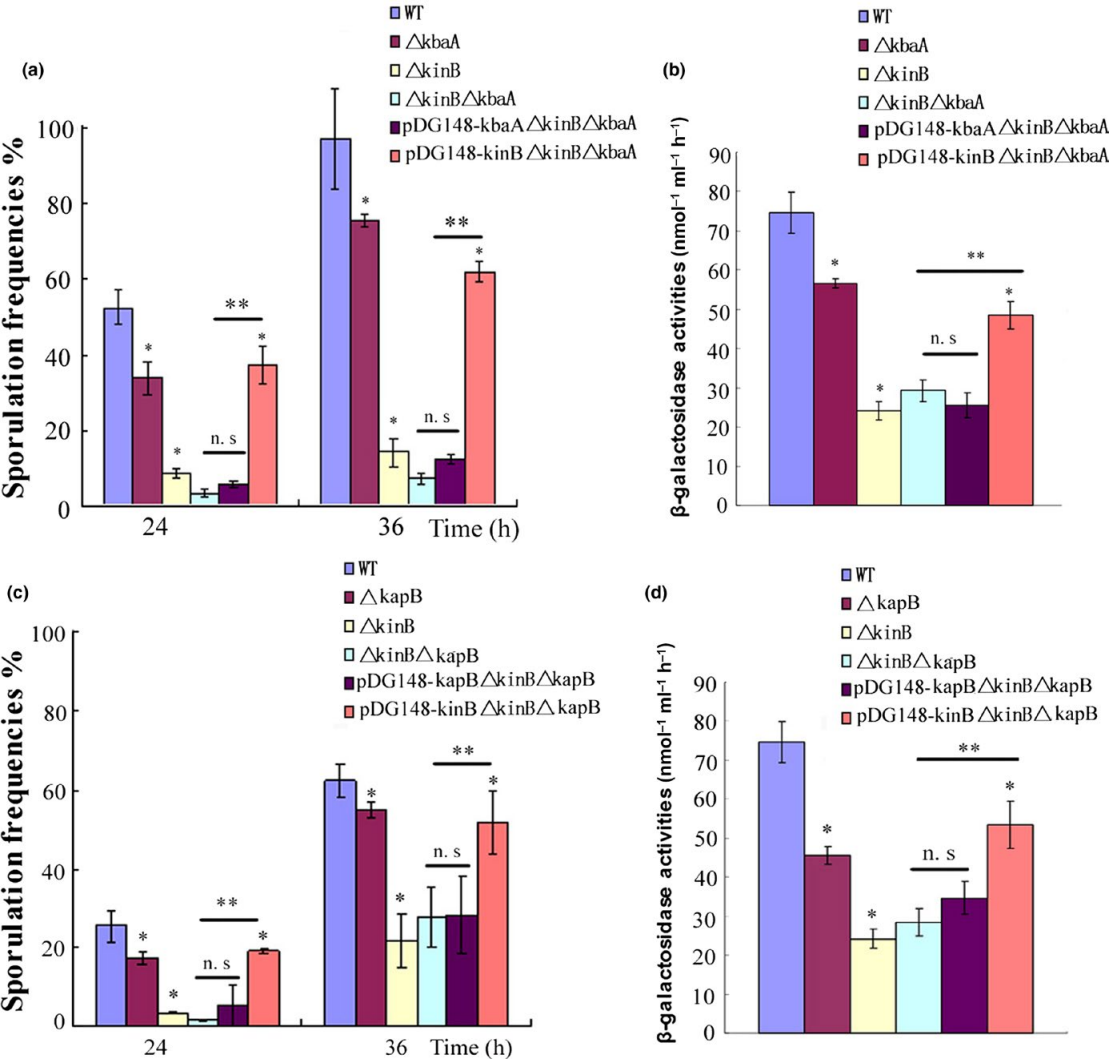
KapB and KbaA function upstream of KinB. (a, b) Comparing the sporulation frequencies and β‐galactosidase activities of Δ*kbaA*, Δ*kinB*, Δ*kinB*Δ*kbaA*,*pDG148‐kinB*Δ*kinB*Δ*kbaA*, and *pDG148‐kbaA* Δ*kinB*Δ*kbaA* mutants in MM medium. In both sporulation and β‐galactosidase activity assays, only the KinB‐complemented strain rescued the phenotype of the double mutant strain Δ*kinB*Δ*kbaA*. (c, d) In the Δ*kinB*, Δ*kapB*, Δ*kinB*Δ*kapB*,*pDG148‐kinB*Δ*kinB*Δ*kapB*, and *pDG148‐kapB*Δ*kinB*Δ*kapB* mutants, the sporulation frequencies and β**‐**galactosidase activities were analyzed. Similarly, only the KinB‐complemented strain could restore the majority of sporulation frequencies and β**‐**galactosidase activities of the double mutant strain Δ*kinB*Δ*kapB*. n.s. *p *≥* *.05, *p < .05 using the wild‐type strain *Bacillus subtilis* 168 as control, **p < .05 using the double mutant strains Δ*kinB*Δ*kbaA* or Δ*kinB*Δ*kapB* as control

We first tried to reveal the relationship between Kba and KinB by comparing the sporulation frequencies of strains Δ*kbaA*, Δ*kinB*, Δ*kinB*Δ*kbaA*,* p*DG148‐*kinB*Δ*kinB*Δ*kbaA*, and *p*DG148‐*kbaA*Δ*kinB*Δ*kbaA* in MM medium. Our data showed that the double mutants of Δ*kinB*Δ*kbaA* had comparable sporulation frequencies as the Δ*kinB* mutant, but were significantly lower than that of the Δ*kbaA* mutant strain. Furthermore, only the complementary expression of KinB (*p*DG148‐*kinB*Δ*kinB*Δ*kbaA*), but not *p*DG148‐*kbaA*Δ*kinB*Δ*kbaA*, could rescue the sporulation defect of the double mutant Δ*kinB*Δ*kbaA*, suggesting that KbaA was localized upstream of KinB in the pathway (Figure 3a). To further confirm our results, we also assayed β**‐**galactosidase activities in those strains. We found that the β**‐**galactosidase activity in the double mutant Δ*kinB*Δ*kbaA* was close to that of the Δ*kinB* mutant. Similarly, the complement of KinB in the mutant Δ*kinB*Δ*kbaA* (*p*DG148‐*kinB*Δ*kinB*Δ*kbaA*) had enhanced β**‐**galactosidase activity (*p* < .05) that was consistent with the results of sporulation frequency (Figure 3b).

Next, to examine the regulatory relationship between KapB and KinB, we analyzed the sporulation frequencies and β**‐**galactosidase activities in Δ*kinB*, Δ*kapB*, Δ*kinB*Δ*kapB*,* p*DG148‐*kinB*Δ*kinB*Δ*kapB*, and *p*DG148‐*kapB*Δ*kinB*Δ*kapB* strains in MM medium. Similar to the results of KabA, our data demonstrated that the double mutant Δ*kinB*Δ*kapB* was close to the Δ*kinB* mutant in both sporulation frequency and β**‐**galactosidase activity (Figure 3c,d). Moreover, *p*DG148‐*kinB*Δ*kinB*Δ*kapB* strain rescued both the sporulation and the β**‐**galactosidase activity of the double mutant (*p* < .05) (Figure 3c,d). Together, our experimental evidences supported the hypothesis that KapB was localized upstream of KinB in the signaling pathway.

**Figure 4:**
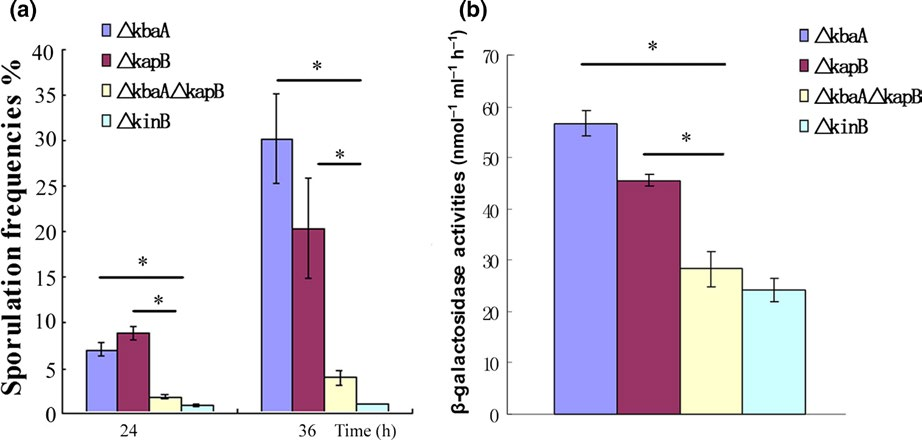
KapB and KbaA regulate KinB through parallel pathways. (a) The sporulation frequencies of Δ*kbaA*, Δ*kapB*, Δ*kbaA*Δ*kapB*, and △*kinB* in MM medium. (b) β‐galactosidase activities of Δ*kbaA*, Δ*kapB*, Δ*kbaA*Δ*kapB*, and △*kinB* in MM medium after grown 36 hr. The double mutant Δ*kbaA*Δ*kapB* had much lower sporulation frequency and β**‐**galactosidase activity than either Δ*kbaA* or Δ*kapB*. n.s. *p *≥* *.05, *p < .05

Since our current data indicated that both KabA and KapB functioned upstream of KinB, we further investigated if KapB and KbaA modulated the sensor kinase KinB via the same or independent pathway(s). After constructing the double mutant strain of Δ*kbaA*Δ*kapB* and analyzing its sporulation frequency and β**‐**galactosidase activity, we found that the double mutant Δ*kbaA*Δ*kapB* represented much lower sporulation frequency and β**‐**galactosidase activity than either Δ*kbaA* or Δ*kapB* (*p* < .05) (Figure 4a,b). Furthermore, it was also noticed that the sporulation frequency and β**‐**galactosidase activity of the Δ*kbaA*Δ*kapB* double mutant were similar to those of the Δ*kinB* mutant. Based on those experimental evidences, it was reasonable to speculate that KapB and KbaA should regulate KinB independently, but not in the same pathway.

### 3.4 Analysis of the protein domains of KapB required in KinB‐dependent sporulation

**Figure 5:**
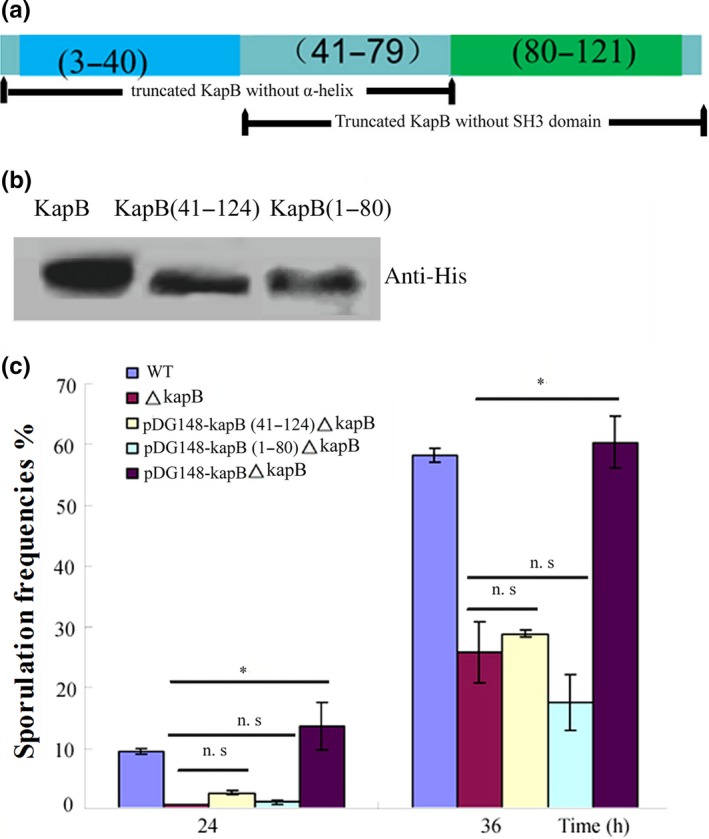
Both the SH3 domain and the α‐helix in KapB are required for KinB‐dependent sporulation. (a) Schematic representation of protein domains in KapB includes a SH3 domain (3–40 amino acids) and an α‐helix (80–121 amino acids). (b) Western blotting assay to determine their expression of the intact or truncated KapB using anti‐His antibody. (c) In the background of Δ*kapB*, the intact KapB or the truncated KapB without either SH3 domain or α‐helix was complemented and expressed and then the sporulation frequencies were determined respectively. Only the complement by intact KapB could rescue the sporulation defect of Δ*kapB*. n.s. *p *≥* *.05, *p < .05

The protein structure prediction of KapB revealed that it had two main domains, an SH3 domain (3–40 amino acids) and an α‐helix domain (80–121 amino acids) (Figure 5a). To verify the roles of these two domains in sensing and transmitting the environmental signal in MM medium, a series of recombinant plasmids overexpressing either the intact KapB or the truncated KapB without either the SH3 domain or the α‐helix were constructed. These recombinant plasmids were then introduced into the Δ*kapB* mutant to obtain *p*DG148‐*kapB*Δ*kapB*,* p*DG148*‐kapB*(41–124)Δ*kapB*, and *p*DG148‐*kapB*(1–80)Δ*kapB*. Since 6× His tag was fused to the KapBs at the C‐terminal, western blotting was employed using the anti‐His antibody to determine their expression. Then it was confirmed the existence of our target proteins as we have expected (Figure 5b). Next, we compared the sporulation frequencies of those three mutants to Δ*kapB* and the wild‐type *B. subtilis* 168 in MM medium. The experimental data demonstrated that the complementary expression of the intact KapB almost restored the sporulation defect of Δ*kapB* (*p* < .05) (Figure 5b). However, both *p*DG148*‐kapB*(41–124)Δ*kapB* and *p*DG148‐*kapB*(1–80)Δ*kapB* showed significantly lower sporulation frequencies than the *p*DG148‐*kapB*Δ*kapB* mutant, which suggested that the expression of those two mutant proteins had little rescue activities on sporulation (Figure 5c). Therefore, both the SH3 domain and the α‐helix domain in KapB should be required for the signal sensing or transduction during sporulation initiation.

## 4 DISCUSSION

Multiple sensor kinases (KinA to KinE) are known to play important roles for initiating phosphorelay and for controlling the level of phosphorylated Spo0A though their roles in phosphorylating Spo0A and turning on the transcription of sporulation‐related genes seemed redundant. Based on the results of analyzing spatiotemporal expression of those histidine kinases, a few researchers suggested that there existed some subtle differences in their stages of action. For example, it had been proposed that the most common kinases KinA and KinB were activated sequentially and KinB was expressed prior to the former (Dartois et al., 1996). The experimental evidences from Hoch research group revealed that the *kinB* gene was expressed at a higher rate than *kinA* during exponential growth and reached a maximum 1.5 hr before *kinA* transcription. Correspondingly, the absence of *kinB* delayed the transcription of *spoIIG* for 1 hr but its ultimate expressional level was not changed significantly (Dartois et al., 1996). At the same time, another study from the same laboratory also showed that in the absence of *kinA* and *kinB*, the phosphorylation mediated by KinC and KinD could also happen at the exponential phase of growth. Thus, it was concluded that all the kinases were expressed at the same stage and the differential activities observed in growth and sporulation might result from differential activation by the signal ligands unique to each kinase (Jiang et al., 2000).

Since sporulation can be initiated via both the external and internal signals, including cell density, nutrient starvation, heat stress, cell cycle, and so on, KinA, KinB, KinC, and KinD have also been suggested to respond individually to distinct stimuli. To the signals sensed by KinB, it was recently shown that the cytosolic potassium and the extracellular oxygen limitation could trigger sliding motility and matrix production in *B. subtilis*, respectively (Grau et al., 2015; Kolodkin‐Gal et al., 2013). The only investigation dealing with the environmental signals in endospore formation illustrated that KinB functioned as the sensor kinase when the bacterial cells were grown in glucose minimal medium (LeDeaux et al., 1995). And our current experimental results based on both sporulation frequencies and β**‐**galactosidase activities confirmed that the depletion of *kinB* led to the serious defects in MM medium, which is generally employed as a sporulation‐inducing medium with limited carbon and nitrogen sources. However, a single nutrient‐limiting factor seems to be insufficient for sporulation because the cells, before sporulation induction in MM medium, had to grow in the nutritional media (e.g., LB medium) to reach a high cell density. When the bacteria cultured only in MM medium throughout the growth and sporulation stages, it also represented an obvious defect on sporulation (data not shown).

On the other hand, the structural characters of KinB having only minimal loop regions and an N‐terminal outside the membrane suggest that it is unlikely to recognize the extracellular signal molecule in the environment directly. So we further determined whether KbaA and KapB could response to the same external signals of nutritional starvation with KinB. Our data validated their roles in the signaling pathway since the absence of those two genes caused the defects of KinB‐dependent sporulation in MM medium. Since *kinB* was mainly epistatic to *kbaA* or *kpaB* and played a predominant role in sporulation initiation in MM medium, it was suggested that KinB should function downstream in the signaling pathway. Furthermore, the double deletion of *kbaA* and *kapB* caused more serious defects than the single gene deletion. Although it is reasonable to hypothesize that KbaA and KapB might have their impacts on sensing the signal of nutrition starvation, those two proteins might only be involved in maintaining KinB as previously reported (Dartois et al., 1996), because the stability and the subcellular localization of KinB had not been observed when KapB was abnormal.

Collectively, our data here support that KinB plays a more important role during sporulation initiation triggered by the nutrient starvation in MM medium than KinA does, which is consistent with the hypothesis that there are different signals unique to each histidine kinase activating their own sensors and the shared downstream phosphorelay. We further analyzed the interrelationship among KinB, KbaA, and KapB in the signaling pathway. But if the environmental cue of nutrient starvation acts through either the cytosolic signal of potassium or the changes in GTP and ATP requires further investigations.

## CONFLICT OF INTEREST

None declared.
